# Comparison of AID users and non-users regarding glycemic control and diabetes distress: A mixed-methods study

**DOI:** 10.1371/journal.pone.0353125

**Published:** 2026-07-24

**Authors:** Alexander Hadj-Abo, Burkhard Gusy, Monika Fleischhauer

**Affiliations:** 1 Division of Prevention and Psychosocial Health Research, Department of Education and Psychology, Freie Universität Berlin, Berlin, Germany; 2 Department of Psychology, MSB Medical School Berlin, Berlin, Germany; Japanese Academy of Health and Practice, JAPAN

## Abstract

**Aim:**

This study compared glycemic control and diabetes distress in individuals with type 1 diabetes using automated insulin delivery (AID) systems versus those not using such systems, addressing a gap in research on psychosocial outcomes of diabetes technologies.

**Methods:**

A sequential mixed-methods design was employed to examine differences between AID users and non-users. A total of 169 individuals with type 1 diabetes completed an online survey assessing HbA1c, total diabetes distress, and its subscales. Quantitative results were complemented by 12 follow-up interviews to provide contextual depth.

**Results:**

The AID group (n = 80) showed significantly lower levels in HbA1c, total diabetes distress, and all subdimensions compared to the non-AID group (n = 89), which showed high CGM use (~94%). The largest effects emerged for *Management Distress* and *Powerlessness*. Interview data suggested that AID systems reduce the cognitive and emotional burden of diabetes self-management, which may explain these differences. Contrary to expectations, group differences in *Hypoglycemia Distress* and *Negative Social Perception* were smaller. This may be explained by the widespread use of continuous glucose monitoring (CGM) systems in the non-AID group, as participants from both groups reported CGM use to be helpful in reducing hypoglycemic events.

**Conclusions:**

AID systems are associated with both, improved glycemic outcomes and enhanced psychosocial well-being. These findings underscore the multifaceted benefits of AID systems and highlight the importance of incorporating psychosocial dimensions into the evaluation of diabetes technologies. The mixed-methods approach proved valuable in capturing both quantitative and experiential aspects, providing a more nuanced understanding of patient experiences.

## Introduction

The global prevalence of diabetes continues to rise, currently affecting an estimated 537 million people worldwide and projected to reach 783 million by 2045 [1]. Alongside increasing prevalence, the health and economic burden of diabetes is substantial and growing. In 2021, an estimated 6.7 million deaths were attributed to diabetes-related causes, with global healthcare costs reaching USD 966 billion [1]. A significant proportion of these costs results from secondary diseases such as depression or anxiety, which are associated with inadequate glycemic control and challenges in diabetes management [2].

Although type 1 diabetes was historically considered a childhood disease, recent evidence indicates a growing number of diagnoses occurring in adulthood [3,4]. Adults with type 1 diabetes therefore represent an increasingly relevant population in both clinical practice and research, and are the focus of the present study.

Technological advances have substantially transformed diabetes management. Automated insulin delivery (AID) systems integrate continuous glucose monitoring (CGM) with continuous subcutaneous insulin infusion (CSII) in a closed-loop architecture that enables algorithm-driven insulin adjustment. In contrast to sensor-augmented pump therapy or other technology-supported regimens without automated insulin modulation, AID systems continuously adapt insulin delivery based on real-time glucose data. By partially automating insulin delivery and glycemic control, they aim to improve metabolic stability while reducing the cognitive and behavioral demands of daily self-management. At the same time, their use introduces technological complexity, including setup and ongoing interaction requirements, which may pose challenges for some users.

The evaluation of diabetes technologies has predominantly focused on glycemic outcomes. HbA1c (reflecting average blood glucose levels over the preceding three months) and Time in Range (TIR, indicating the percentage of time glucose remains within the target range) have become central indicators of metabolic control [5]. An HbA1c value of approximately 5.5–6.5% (i.e., percentage of total hemoglobin that is glycosylated) is generally considered within the optimal physiological range for long-term glycemic control [6,7]. Existing evidence indicates that technological advances in insulin delivery and glucose monitoring – ranging from insulin pens to CSII and CGM – consistently improve glycemic control and treatment adherence compared to conventional injection-based approaches. These findings are supported by multiple randomized controlled trials and meta-analyses, which collectively demonstrate superior HbA1c outcomes among users of these technologies [8–11].

More recent evaluations of AID systems indicate additional improvements in glycemic outcomes beyond those achieved with CGM and CSII alone. Meta-analytic evidence from randomized controlled trials demonstrates consistent reductions in HBA1c and increased TIR among AID users [12]. However, many of these studies were conducted in controlled clinical settings, often excluding participants with comorbidities or suboptimal glycemic control, which may limit the generalizability of the findings. To address this limitation, real-world analyses encompassing large and more diverse populations have been conducted. These studies consistently report clinically meaningful improvements in both HbA1c and TIR across broad patient groups, confirming the effectiveness of AID systems outside of tightly controlled trial environments [13].

While these findings highlight the clinical benefits of AID systems, diabetes management extends beyond metabolic outcomes. Unlike many other chronic conditions, diabetes requires continuous daily self-management, placing a substantial cognitive and emotional burden on those affected [14]. Additionally, the adverse effects of poor glycemic control often develop gradually, which can reduce the perceived urgency of maintaining strict glycemic control. Consequently, for many individuals, the psychological demands of intensive therapy can rival or even outweigh the burden associated with suboptimal glucose control [13].

Accordingly, patient-centered perspectives have gained increasing importance in both research and clinical practice [15]. Beyond improving metabolic outcomes, reducing treatment burden and enhancing quality of life are now recognized as central goals. From a health economics standpoint, this is highly relevant, as the German Diabetes Society’s 2024 Health Report notes that patient time costs (i.e., the monetized value of time spent managing the disease) now exceed direct medical costs [2].

Patient-reported outcomes (PROs) provide a means of capturing these broader impacts. Although studies suggest that technology-supported therapies may improve PROs [12,16], such outcomes are frequently treated as secondary endpoints, and comparability across studies remains limited due to heterogeneous measurement approaches. Moreover, many of these measures focus on technology acceptance or specific concerns like hypoglycemia anxiety, while more comprehensive psychosocial constructs are less frequently examined [16].

One such psychosocial key construct is diabetes distress, defined as the emotional burden associated with the ongoing demands of diabetes self-management [17]. Distinct from depression [14], diabetes distress reflects a core aspect of the lived experience of the disease and is reported by approximately 20–40% of individuals with type 1 diabetes [18,19]. Importantly, higher levels of diabetes distress are associated with poorer glycemic control [20], suggesting potential bidirectional relationships between psychological burden and metabolic control.

Diabetes distress in type 1 diabetes encompasses multiple dimensions, including feelings of powerlessness, management-related strain, hypoglycemia-related concerns and social distress [18,21]. These dimensions provide a differentiated framework for examining how diabetes technologies may influence psychosocial well-being.

Given their automation and potential to reduce cognitive demands, AID systems may mitigate specific aspects of diabetes distress. By automating insulin delivery, they can reduce the burden of day-to-day disease management, minimize dosing errors, and lower the frequency of extreme glycemic events. Qualitative research supports this assumption. Suttiratana et al. [22] reported that AID users experienced lower mental stress, felt more supported in managing their condition, and reported fewer hypoglycemic episodes, especially at night. Similarly, Polonsky et al. [23] observed significant reductions in the corresponding dimensions of the Type 1 Diabetes Distress Scale, that is in *Powerlessness*, *Management Distress*, and *Hypoglycemia Distress,* after three months of AID use. Additionally, a comparative study reported differences in the *Powerlessness* dimension between AID users and sensor-supported CSII users [24].

Another advantage mentioned by patients in the qualitative study by Suttiratana et al. [22] was the discretion offered by AID systems. This suggests that AID users may also report lower scores in the *Negative Social Perception* dimension of Diabetes Distress compared to non-users.

However, current evidence on AID systems remains primarily focused on glycemic outcomes, while their impact on diabetes distress – particularly across its multidimensional structure – remains insufficiently understood in adult populations with type 1 diabetes. Existing studies often examine isolated psychosocial outcomes or rely on controlled trial settings, limiting comparability and real-world application.

The present study addresses this gap by comparing adults using AID systems with non-users in terms of both glycemic control and diabetes distress. By jointly assessing a clinical indicator and patient-reported outcomes, the study aims to provide a more comprehensive and patient-centered evaluation of diabetes technology. In doing so, it responds to calls for integrating lived experiences into the assessment of technological interventions in diabetes care [16].

Based on the literature outlined above, the study tested the following hypotheses:

H1: A higher degree of automation is associated with improved glycemic control.

H2: A higher degree of automation is associated with lower overall diabetes distress.

H3: Differences between groups are more pronounced in the dimensions Management Distress, Hypoglycemia Distress, Powerlessness, and Negative Social Perception than in other dimensions of diabetes distress.

To complement the quantitative analyses, qualitative interviews were conducted to gain deeper insight into group differences in diabetes distress. In particular, the study explores whether the automation of glucose management may free cognitive resources that can be redirected toward other aspects of diabetes self-management, reflecting a potential spillover effect.

## Method

The presented hypotheses employed a sequential explanatory mixed-method design, in which quantitative data collection and analysis were followed by qualitative interviews to further explore and contextualize the quantitative findings. The data were nested, meaning that the interviewed participants were drawn from the same sample that participated in the quantitative survey. The criteria for selecting interview participants are described in the qualitative methods section.

### Quantitative part

#### Procedure.

Participants were eligible for inclusion if they were of legal age and had been diagnosed with type 1 diabetes for at least six months at the time of the survey. Data collection took place between 14/11/2023 and 29/02/2024. Participants were recruited through several channels. Flyers were distributed during diabetes consultation hours at Charité Mitte. In addition, a call for participation was sent to members of the online panel of the Diabetes Centre Bad Mergentheim. Further recruitment took place within diabetes-related online communities such as the Looper Group Berlin, and through social media invitations by diabetes influencers on platforms including Instagram and TikTok.

The online survey was conducted via the survey platform Tivian. Before beginning the survey, participants received written information about the study aims and the use of the collected data. Participants then generated a personalized code word based on a predefined formula. This code was used to enable the merging of quantitative and qualitative data and to allow participants to request the deletion of their data if desired. Participation in the survey started only after informed consent had been obtained. The study was conducted in accordance with the Declaration of Helsinki (revised version) and the ethical standards of the German Psychological Association. The ethics committee of the MSB Medical School Berlin approved the study procedure and declared it to be ethically unobjectionable (committee’s reference number: MSB-2023/146).

#### Participants.

The total sample consisted of N = 172 participants, including 83 users of AID systems and 89 participants who did not use AID systems. Three participants who identified as diverse or did not report their gender were excluded from statistical analyses due to the inclusion of gender as a covariate and the small size of this subgroup, which did not allow meaningful statistical analysis. The final analytical sample therefore consisted of N = 169 participants (AID: n = 80, non-AID: n = 89). A detailed description of this sample is presented in the Results section.

Participants were categorized into the AID group if they reported using automated insulin delivery systems integrating continuous glucose monitoring (CGM) with continuous subcutaneous insulin infusion (CSII) and algorithm-driven insulin adjustment. The non-AID group included different combinations of participants using CGM or punctual blood sugar testing with test strips and multiple daily injections (MDI) or CSII, as long as there was no algorithm-driven insulin adjustment.

#### Measures.

***Glycemic control.*** Glycemic control was assessed using self-reported glycohemoglobin (HbA1c) values. HbA1c reflects average blood glucose levels over the previous three months and is considered the primary indicator of glycemic control during this period [5].

***Diabetes distress.*** Diabetes distress and its dimensions were measured using the German version of the Type 1 Diabetes Distress Scale (T1-DSS) [21]. The T1-DSS consists of 28 items, which form a total score and seven sub-dimensions:

1   Powerlessness describes a generalized feeling of discouragement regarding one’s diabetes (5 items; e.g., “The feeling that no matter how hard, my diabetes will never be good enough.”)
2 *Management Distress* refers to disappointment with one’s own efforts to self-manage diabetes (4 items; e.g., “The feeling that I am not paying as much attention to my diabetes as I should.”)
3 *Hypoglycemia Distress* describes concerns about hypoglycemic events (4 items; e.g., “The feeling that I can never be safe from the possibility of severe hypoglycemia.”)
4 *Negative Social Perception* refers to worries about possible negative judgements from others (4 items; e.g., “The feeling that I have to hide my diabetes from others.”)
5 *Eating Distress* is the fear that one’s eating behavior is getting out of control (3 items; e.g., “The feeling that thoughts about food and nutrition determine my life.”)
6 *Physician Distress* describes disappointment with healthcare professionals’ treatment (4 items; e.g., “The feeling that I am not getting the support I really need from my doctor in dealing with diabetes.”)
7 *Family and Friends Distress* reflects the perception that family members and friends focus too much on the disease (4 Items; e.g., “The feeling that my family and friends are making a bigger deal out of my diabetes than they should.”)

The items are rated on a six-point Likert scale, ranging from 1 (not a problem at all) to 6 (a very big problem). Scores are calculated as the mean of the respective items. According to established cut-off values, mean scores below 2.0 indicate little or no distress, scores between 2.0 and 2.9 indicate moderate distress, and scores of 3.0 or higher reflect severe distress. Scores above 2.0 are considered clinically significant [25].

#### Statistical analyses.

All statistical analyses were conducted using IBM **SPSS** Statistics 31. First, correlation analyses were performed to examine the bivariate relationships between the T1-DSS total score, its subscale scores, HbA1c, and socio-demographic variables. This analysis also served to identify possible confounding variables that could be included as covariates in subsequent analyses.

To examine mean differences between AID users and non-users in terms of glycemic control, overall diabetes distress, and the individual distress dimensions, the assumptions for ANOVA and ANCOVA models were assessed. Normality was evaluated by the Shapiro-Wilk test, which indicated deviations from normality for the dependent variables (each *p* < .2). However, as no outliers were detected and skewness and kurtosis values were within the acceptable range of ±2, the distributions were considered approximately normal [26,27]. Since the assumption of homoscedasticity was violated in some analyses, as indicated by significant results of the Levene and White tests, heteroscedasticity-consistent robust standard errors (HC3) were applied. This approach provides more reliable estimates when variances are unequal. An a priori power analysis using G*Power (version 3.1.9.7) for an ANCOVA (fixed effects, main effects and interactions) with one between-subject factor (AID vs non-AID) and five covariates indicated that a total sample size of *N* = 128 would be required to detect a medium effect (*f* = 0.25) with *α* = .05 and a power of 0.80. The final analyzed sample (*N* = 169) exceeded this requirement.

### Qualitative part

#### Sample and recruitment.

At the end of the online survey, participants were asked whether they would be willing to participate in a follow-up interview. Participants who agreed were asked to provide their email address for further contact. Email addresses were stored separately from the survey data to ensure that quantitative responses could not be linked to personal information. Of the 169 participants, 54 agreed to be contacted again, and 30 responded to the interview invitation. A total of 12 interviews were conducted, with six participants in each group (AID users and non-users). Within the last two interviews, only marginal or no new information was obtained, and it was therefore assumed that data saturation had been reached. Detailed descriptive characteristics of the interview sample are presented in the Results section.

### Interview procedure

Based on the results of the quantitative analysis, an initial semi-structured interview guide was developed. The guide was continually refined and expanded during the interview phase as new themes emerged.

Interviews were conducted in different formats: One of the 12 interviews was conducted in person, five by telephone, and six via the Microsoft Teams video call platform. All interviews were audio-recorded and manually transcribed. Before the interview began, participants were informed about the recording, storage, transcription, pseudonymization of personal data, and their right to withdraw from the study at any time. Participants gave their consent to the recording before the interview started.

Participants also recreated the code word they had generated during the quantitative part, which enabled the merging of quantitative and qualitative data while maintaining anonymity. To further ensure anonymity, email correspondence was deleted on the day of the interview, and no real names were included in the transcripts or recordings.

## Results

### Quantitative results

#### Sample characteristics.

By definition, all individuals in the AID group used CSII and CGM systems, whereas in the non-AID group approximately one third (34.8%) used CSII and the majority relied on insulin pens (65.2%). Despite these differences in insulin delivery methods, CGM use was also highly prevalent in the non-AID group (94.4%), with only 5.6% relying on blood glucose test strips. Overall, both groups demonstrated a high level of diabetes technology use.

As shown in Table 1, the AID and non-AID groups were broadly comparable with respect to age, gender, and duration of diabetes (*p* > .05). However, significant differences emerged in educational characteristics, with AID users having a higher level of education than non-users (*p* = .008).

**Table 1:** Sample characteristics.

	Total sample (N = 169)		AID system (n = 80)		No AID system (n = 89)		
	*M (SD)*	*Range*	*M (SD)*	*Range*	*M (SD)*	*Range*	*p-value*
Age (in years)	36.2 (16.5)	18-80	37.1 (15.5)	18-69	35.3 (17.3)	18-80	.468
Years since diagnosis	17.4 (13.1)	0.7-65	19.2 (12.8)	1.0-56	15.8 (13.3)	0.7-65	.095
	***N***	***%***	***N***	***%***	***N***	***%***	
Gender							.136
female	133	78.7	59	73.8	74	83.1	
male	36	21.3	21	26.2	15	16.9	
Education							.008
PhD	5	3.0	4	5.0	1	1.1	
University degree	74	43.7	42	52.5	32	36.0	
University entrance qualification/ vocational training	62	36.7	23	28.7	39	43.8	
Intermediate school	23	13.6	9	11.3	14	15.7	
Lower secondary school	5	3.0	2	2.5	3	3.4	

*Notes.* For education, the p-value refers to a dichotomized variable (higher education: university degree or PhD vs. lower education).

#### Descriptive statistics and intercorrelations of the study variables.

As depicted in Table 2, the internal consistencies of the Diabetes Distress total scale and most subscales were good to excellent (Cronbach’s α = 0.78–0.93) and comparable to previous German and English validation studies [18,21]. The subscale Eating Distress showed somewhat lower internal consistency (Cronbach’s α = 0.67) which is likely related to the smaller number of items and their potentially greater heterogeneity compared to the other subscales.

**Table 2:** Means, standard deviations, internal consistencies, and intercorrelations among study variables.

Variable	*M*	*SD*	1	2	3	4	5	6	7	8	9	10	11	12
1. DDS-Total Score	2.72	0.90	.93											
2. Powerlessness	3.67	1.20	.82^***^	.82										
3. Management-Distress	2.57	1.17	.73^***^	.55^***^	.78									
4. Hypoglycemia Distress	2.53	1.23	.71^***^	.52^***^	.47^***^	.79								
5. Negative Social Perception	2.27	1.24	.70^***^	.52^***^	.39^***^	.42^***^	.83							
6. Eating Distress	3.34	1.26	.74^***^	.67^***^	.50^***^	.48^***^	.40^***^	.67						
7. Physician Distress	2.36	1.37	.65^***^	.40^***^	.46^***^	.31^***^	.45^***^	.35^***^	.90					
8. Friend and Family Distress	2.20	1.19	.64^***^	.34^***^	.43^***^	.50^***^	.43^***^	.39^***^	.32^***^	.83				
9. HbA1c	6.96	1.15	.23^**^	.17^*^	42^***^	.14	.08	.19^**^	.07	.07	—			
10. Age	36.17	16.46	-.25^**^	-.21^**^	-.32^***^	-.09	-.30^***^	-.19^**^	-.05	-.12	-.21^*^	—		
11. Time since first Diagnosis	17.44	13.15	-.26^***^	-.21^**^	-.20^**^	-.23^**^	-.26^***^	-.21^**^	-.07	.10	-.04	.52^***^	—	
12. Gender ^a^	—	—	-.19^*^	-.20^**^	-.13	.01	-.17^*^	-.19^*^	-.16	-.04	-.02	.37^**^	.24^**^	—
13. Education ^b^	—	—	-.20^**^	-.17^*^	-.13	-.29^***^	-.12	-.16^*^	.03	-.18^*^	-.32^***^	.33^***^	.26^**^	.03

*Notes.* ^a^ N = 169 (excluding diverse individuals and non-responders), ^b^Spearman correlations were conducted. *M* = Mean, *SD* = Standard Deviation, Cronbach’s *α* coefficients are presented on the diagonal, DDS = Diabetes Distress Scale. ^*^p < .05, ^**^p < .01, ^***^p < .001.

Correlation analysis indicated that age was negatively associated with several distress variables and HbA1c, suggesting lower distress and better glycemic control with increasing age. Gender was significantly associated with the subscales Powerlessness and Eating Distress suggesting that women experience more distress than men. In addition, education was negatively correlated with HbA1c, the total distress score, and several subscales, with higher education being associated with better glycemic control and lower distress. Based on these empirical associations as well as prior evidence indicating that sociodemographic factors are related to both diabetes outcomes and the uptake of diabetes technologies [28], age, gender, and education were included as covariates in subsequent analyses to account for potential confounding. To facilitate inclusion in the models and ensure sufficient group sizes, education was dichotomized (university entrance qualification or lower, *n* = 90, vs. university degree or higher, *n* = 79).

#### Group differences: AID vs. non-AID group.

Results of the ANCOVA analyses are summarized in Table 3. With regard to glycemic control, the AID users showed significantly lower HbA1c values (*M* = 6.68, *SD* = 0.74) compared to non-users (*M* = 7.21, *SD* = 1.38), indicating better glycemic control (F(164) = 5.81, *p* = .017, partial *η²* = .034).

**Table 3:** ANCOVA results for glycemic control and diabetes distress (adjusted for age, gender, and education).

Dependent variable	*F*	*p*	*p* (FDR)	*partial η²*	*M (SD)* AID use (n = 80)	*M (SD)* No AID use (n = 89)
HbA1c						
group	5.81	**.017**	**.026**	.034	6.68 (0.74)	7.21 (1.38)
age	2.58	.110		.016		
gender	0.11	.740		<.001		
education	9.02	**.003**		.052		
T1-DDS-Total Score						
group	14.40	**<.001**	**.002**	.081	2.42 (0.78)	2.99 (0.92)
age	2.85	.094		.017		
gender	0.33	.567		.002		
education	2.04	.155		.012		
Powerlessness						
group	11.204	**.001**	**.003**	.064	3.31 (1.15)	4.01 (1.15)
age	3.364	.068		.020		
gender	2.491	.116		.015		
education	2.196	.140		.013		
Management-Distress						
group	12.76	**<.001**	**.002**	.072	2.22 (1.03)	2.88 (1.21)
age	8.36	**.004**		.048		
gender	0.22	.641		.001		
education	0.37	.545		.002		
Hypoglycemia Distress						
group	4.03	**.046**	**.046**	.024	2.28 (1.17)	2.76 (1.24)
age	0.01	.960		<.001		
gender	0.59	.442		.004		
education	8.78	**.003**		.051		
Negative Social Perception						
group	5.64	**.019**	**.024**	.033	2.01 (1.07)	2.51 (1.34)
age	8.66	**.004**		.050		
gender	<0.01	.976		<.001		
education	0.18	.668		.001		
Eating Distress						
group	4.47	**.036**	**.041**	.027	3.08 (1.23)	3.58 (1.24)
age	2.11	.148		.013		
gender	2.27	.134		.014		
education	1.40	.239		.008		
Physician Distress						
group	7.88	**.006**	**.013**	.046	2.05 (1.20)	2.63 (1.46)
age	0.07	.798		<.001		
gender	0.97	.327		.006		
education	1.15	.286		.007		
Friend and Family Distress						
group	5.89	**.016**	**.029**	.035	1.93 (0.88)	2.44 (1.37)
age	0.04	.834		<.001		
gender	<0.01	.971		<.001		
education	2.96	.087		.018		

*Notes.* N = 169. Degrees of freedom for all tests: df = (1, 164). AID = automated insulin delivery; T1-DDS = Type 1 Diabetes Distress Scale. M (SD) values are presented for descriptive purposes and refer to unadjusted group means. Robust standard errors (HC3) were used to account for potential heteroscedasticity. False discovery rate (FDR; Benjamini–Hochberg procedure) correction was applied to all group effects across outcomes. p-values for covariates are reported unadjusted. Statistical significance is based on two-tailed tests.

AID users also reported significantly lower overall diabetes distress (*M* = 2.42, *SD* = 0.78) than non-users (*M* = 2.99, *SD* = 0.92; F(164) = 14.40, *p* < .001, partial *η²* = .081). Across subscales of the T1-DSS, a consistent pattern emerged, with AID users reporting significant lower distress levels in all domains. The largest effects were found for Management Distress (*η²* = .072) and Powerlessness (*η²* = .064), while the smallest group differences were observed for the dimensions Hypoglycemia Distress (*η²* = .024), Eating Distress (*η²* = .027) and Negative Social Perception (*η²* = .033). To control for multiple testing, a Benjamini-Hochberg correction (FDR) was applied. All group differences remained significant after correction.

### Qualitative part

#### Interview approach.

Semi-structured interviews were conducted using an interview guide that was developed based on the quantitative findings, with the aim of further exploring and explaining observed group differences in diabetes distress. Questions were formulated in an open and group-independent manner to avoid leading participants toward predefined assumptions. Rather than explicitly referring to AID systems, the interview focused more broadly on the role of the diabetes-related technologies in daily management.

Following initial questions on technology use and its general impact on their diabetes management, the interview was structured around the dimensions of diabetes distress that had shown relevant patterns in the quantitative analyses. For each dimension, open-ended questions were asked and followed by prompts for clarification or elaboration where necessary. Examples of such open questions included “How would you describe your relationship with your doctor?,” “Do you feel that your friends and family make a bigger issue out of your condition than you would like?,” and “How do you feel when you think about hypoglycemia?.”

If diabetes technologies were not mentioned spontaneously, participants were specifically asked whether and how the respective experience had changed due to their use. For selected dimensions (e.g., Negative Social Perception and Management Distress), additional follow-up questions explored perceived changes over time and the role of age. The order of topics was flexible and adapted to the flow of the conversation.

#### Integration of quantitative and qualitative findings.

Table 4 presents a joint display integrating quantitative and qualitative findings across diabetes distress dimensions. Overall, the interviews provided contextual explanations for the observed quantitative group differences in diabetes distress and glycemic control, thereby enabling integrated interpretation across methods.

**Table 4:** Joint display integrating quantitative and qualitative findings on AID use and non-use across diabetes distress dimensions.

Quantitative results	Key qualitative results to explain the quantitative results	Sample quotations
The distress values of AID users on the *Powerlessness* dimension are lower than the values of non-users.	AID systems correct and react to small errors that, if left unaddressed, can lead to serious hypoglycemic or hyperglycemic events, that sometimes require hours of treatment, especially in the case of hyperglycemia. The preventative nature of AID systems can therefore help to reduce frustration and feelings of powerlessness.	T8AID: “I also have ADHD, so it’s relatively relaxing that if I snack somewhere and forget to give insulin or something like that, that it doesn’t drag on forever, that it goes down again. That’s because if I’ve just eaten something beforehand and I’ve overlooked the fact that I had to give insulin or I’ve misjudged a bit and then come out a bit higher, then I’ve been dealing with it for ages, if you haven’t seen it and so the system does that by itself.” T4NAID: (on the question of whether there is a feeling of powerlessness) “So only in such, let’s say, crisis phases, where you’re really just tinkering around for hours and don’t know what the problem is.” T9AID: “[...] the active component has only been added by the AID system, which tries to intercept everything at an early stage before it even gets out of hand.”
The distress values in the AID group on the dimension *Management Distress* are lower than the values in the non-AID group. Age has a significant influence on the distress dimension.	AID systems allow more flexibility in the daily routine and reduce the effort required for diabetes management, for example during physical activity or sport, by automatically adapting the insulin balance to situational changes in sugar levels. As they get older, those affected learn coping mechanisms, develop resilience, and accept their illness as part of their lives.	T3NAID: “But this simple desire to go out of the front door with the front door key and go for a walk wherever I want without thinking beforehand, will I be gone for a long time, will I go for a short time or do I meet someone on the way and then just talk to each other without obligation and not think I have to go on or I have to go back or I don’t know my value[...] So it’s just the dependency and that nothing is spontaneous.” T6AID: “They (AID system) help me in my management in that I have more space in my head to take care of other things or to completely forget about sugar for a while, because I can simply rely on the fact that things are usually going well. For example, when I go for a walk in the woods with my dog or something like that, there are phases where I used to have to look pretty closely to see if I was doing well in the range and so on. Now, of course, I also check when I set off, but when I do, it’s often the case that I don’t think about it for an hour and a half and then only look up after the walk and see, “Ah ok, I didn’t have hypoglycemia, I didn’t notice anything and the system took care of it quite well.” T9AID: “I have simply accepted the fact that I have a chronic illness and I try to make the best of it, to really be able to enjoy the beautiful moments in life and I think that is a basic inner attitude that you develop, which helps you to deal with sometimes difficult situations and it just helps to get back up, carry on and make sure that you are prepared for future situations from this wealth of experience.”
The distress values of the AID users on the dimension *Hypoglycemia Distress* are lower than the values for non-users, but the group differences are smaller than assumed.	CGM systems, used by almost all participants in the non-AID group, are perceived as a safety net against hypoglycemia due to their high data density, trend displays, and alarm functions. This indicates that the CGM system itself already reduces the perceived burden of hypoglycemia for participants in this group. The difference is nonetheless there, as AID systems prevent many of the hypoglycemias before they even manifest themselves, further reducing the burden and fear of hypoglycemia. AID systems are proactive and CGM systems are merely reactive.	T1NAID: “I have definitely had fewer hypoglycemic episodes since (using CGM), I would say, because I simply see them coming earlier due to falling curves.” T10NAID: “The good thing about the app, i.e., the sensor, is that I can set the alarm earlier and then I already know, sometimes 15 minutes earlier, whether hypoglycemia is coming [...]; that I no longer wake up at night due to hypoglycemia, but that the device wakes me up early [...] where I can still think clearly [...] but at a very low, yes I don’t know how to explain it. The vision is just a bit tunnel-like. You can just feel it. Yes, I prefer it when I wake up earlier and it’s definitely an extremely big help.” T5NAID: “Yes, yes, I’m very scared because it used to put me out of action for quite a long time, before the sensor, and now I don’t have the sensor set quite so tightly and it warns me in good time and that’s good. That’s great, even at night, because then it doesn’t affect you as much because you can intervene beforehand.” T2AID: “As good as not at all, as I said, because I can see the values every 5 minutes and can react or at night my loop system, which regulates itself, I’m no longer afraid (of hypoglycemia).” T12AID: “Well, I definitely notice a big difference, so I’ve had 2 hypoglycemic episodes in the 3 months I’ve had the AID system, so it’s a lot less now.”
The distress values on the dimension *Negative Social Perception* are lower than the values in the non-AID group, but the group differences are smaller than hypothesised. Age has a significant influence on the distress dimension Negative perception of diabetes in the social environment.	Diabetes technologies make the disease less visible and less noticeable to others, as blood glucose measurements from fingertip tests or injections in public places can be avoided. Glucose levels and insulin delivery can be monitored and controlled via mobile phones. It should be noted, however, that AID systems were not directly mentioned in the interviews as a reason for reduced exposure to potential negative perceptions. Instead, CSII and CGM systems were referenced. With increasing age, outward appearances become less important to those affected, and as mentioned earlier, they accept the illness as a part of themselves.	T3NAID: “I try to do it beforehand, for example when I go out to eat and when I’m in a restaurant, pub or wherever, I do it in the toilet. I go there where I’m protected from the eyes of others and I don’t like the idea of sitting in a restaurant and then having to inject myself with insulin. You also have to make yourself free, I don’t like it that way” T5NAID: “No, but it’s easier now thanks to the technology, I used to be ashamed of measuring in public and I used to just leave it alone and accept bad results.” T7NAID: “[...] in my work I’ve always tried to wear a T-shirt so that you can’t see it. yes, now, -I don’t work anymore -now I don’t care.” T11AID: “[...] well, I was younger then and also more vain and I didn’t want people to see that [...] and when I was in my 40s, I didn’t care. It’s part of me and everyone can see it and that’s just how my attitude to it changes. But I think that also has to do with age.”
The distress values on the dimension *Eating Distress* are lower in the AID group than the values in the non-AID group	Those affected rely on the AID systems to correct small and large miscalculations, thereby limiting themselves less. In contrast, those without AID systems expend more mental energy on accurate calculations and units.	T5NAID: “Well, it’s characterized by that, because you have to think all day about what you’re eating, how much you’re eating, when you’re eating, I think that’s stressful. You can eat anything, but you can’t just do it on the spur of the moment and that’s stressful.” T11AID: “If I’m at a birthday party and there’s a birthday cake, then I eat that too, because it’s somehow nicer and I wouldn’t deny myself that or say, oh, I’m not allowed to do that [...]. I always have the feeling that the pump regulates itself, so if it’s very high and I don’t look at it myself, it corrects itself. Yes, that’s, so that’s when I think ok, the auto-correction will think of it so that I can come down again.”
The distress values on the dimension *Physician Distress* are lower than the values in the non-AID group	A heterogeneous picture emerges in the interviews with regard to attitudes towards the treating doctor. For example, some of the AID users experience their visits to the treating doctor as a duty with no greater added value, but this does not pose a problem due to their good attitude. However, no correlation was found in the quantitative data between glycemic control and this dimension of distress (*r* = .05). Others describe practitioners as being open to and encouraging the uptake of new technologies in the past. Thus, the reduced distress might have already existed before the adoption of the technologies, because doctors who recommend such technologies may trigger less distress in patients by offering hopeful future options.	T6AID: “He’s nice and all, and the others were all great too, but they’re just happy with the way things are going. I mean, I’m actually happy too, I don’t need any extreme help or anything, because I’m very independent and that’s why I only go there for check-ups and to get insulin.” T11AID: “The doctor, the diabetologist, hardly plays a role at all, I see him once a year, he’s also older and he only ever sees me and says: yes, it’s going well and well, these are short appointments, he’s not really an important contact person. Otherwise I just go to the diabetes consultant every three months[...] That’s also a compulsory appointment, I always go there, dutiful as I am, but it doesn’t really have any added value for me. I now think she’s telling me things that I didn’t know before [...].” T2AID: “Yes, it was simply the case that he led me there, so to speak, both to the CGM and to the pump. He worked on me for a while. I was sceptical about it at the beginning and he led me there and I’m grateful to him for leading me there and I also ask myself personally, why wasn’t I open to it earlier?”
The distress values on the dimension *Family and Friends Distress* are lower in the AID group than the values in the non-AID group	The burden on family and friends is perceived to be lower, as the challenges of the illness are reduced through the use of the systems. Consequently, the concern of others is diminished, which in turn lowers the distress friends and family project onto the individuals living with the disease.	T9AID: “Well, I notice from the feedback, especially from family and friends, how much everyone involved realizes, that you benefit from such a system and how much less stressful moments occur and that alone is an absolute moment of happiness [...]” T2AID: “With friends or now with my wife, who have really experienced severe derailment several times, and also with friends, they are more likely to talk to me about it, but it has now improved in recent years due to the good settings associated with the CGM and the pump, and it is no longer a big issue, even in my close circle of friends, because it is simply yes, because I have it well under control, because it is simply going well.”

*Notes.* Integration of quantitative results regarding distress scores and covariates and qualitative results applied for the purposes of complementarity and development. T1-T12 = participants in order of time of interview, AID = use of an AID system, NAID = no use of an AID system

Across dimensions, participants’ narratives reflected the quantitative patterns observed between AID users and non-users. Lower *Powerlessness* in the AID group was reflected in descriptions of automated correction of glucose fluctuations and reduced persistence of dysglycemic episodes. Reduced *Management Distress* was mirrored in accounts of increased flexibility and a lower cognitive load in daily diabetes management. With regard to *Hypoglycemia Distress*, participants in both groups described a continuous glucose monitoring as an important source of safety, particularly due to alarm functions and trend information. AID users additionally emphasized the benefit of automated insulin adjustments in preventing hypoglycemic events. Lower *Eating Distress* among AID users was reflected in reports of reduced need for precise carbohydrate calculations and greater flexibility in food intake. In contrast, non-AID users described a higher cognitive burden related to continuous planning and calculation. For *Negative Social Perception*, participants across both groups reported that modern diabetes technologies can reduce the visibility of the condition in everyday life, particularly through the use of CGM and insulin pumps. However, AID systems were not specifically identified as contributing additional advantages in this regard. *Family and Friends Distress* was reflected in participants’ perceptions that improved glycemic management reduced the perceived burden on close others and decreased their involvement in daily disease management. Finally, *Physician Distress*, was characterized by heterogeneous experiences, ranging from low perceived relevance of routine consultations to positive evaluations of supportive healthcare providers.

#### Additional emerging themes.

Beyond the predefined distress dimensions, several additional themes emerged inductively during the interviews. When a topic was mentioned multiple times and considered interesting, it was explored in the subsequent interviews.

One recurring topic was the perceived dependence on diabetes technologies. While most participants did not describe their use in terms of dependency, many emphasized the substantial benefits and expressed that they would not want to return to previous treatment methods. For example, interviewee nine (AID user) said: “So the system itself gives me so much added value in every respect physiologically, mentally, in terms of quality of life and so on, that I would find it very, very, very, very difficult to do without it, whether I can describe it as an addiction, I don’t know. I can certainly cope with pen therapy again from one day to the next, but it would just make a lot of things more complicated, more complex, more difficult.”

Another theme related to the reduced visibility of diabetes due to increasing technologization. While this was often experienced as relieving, some participants reported that their condition received less attention or understanding from others. For example, when asked “Do you feel that your friends and family make a bigger deal of your illness than you would like?” several participants responded that they actually felt the opposite. Respondent five, for instance, said: “No, not really, on the contrary, they don’t really realize it and I often think a bit of support would be appropriate..” In this context, participants expressed a desire for greater recognition of the ongoing effort required to manage the disease even when using AID systems.

Participants also reported challenges associated with the technologies, including technical issues (e.g., signal loss, occlusion) and physical reactions such as skin irritations. In case of interviewee 12 (AID user), dissatisfaction with a specific system was described, including perceived negative effects on glycemic control, leading to changes in devices. Interviewee four (non-AID user) reported frequent occlusions and supplemented pump therapy with long-acting insulin as an additional safety measure.

Finally, continuous glucose monitoring was described as highly beneficial across both groups. However, some participants reported that the constant availability of glucose data initially led to increased stress and over-monitoring, which decreased over time as they adopted to the technology. For example, interviewee six (AID user) explained: “I also had to learn how to deal with the fact that you constantly saw the value and also constantly saw the progression, which you didn’t see otherwise. [...] that stressed me out a bit at the beginning and then I also tended to overreact at the start when it shot up. [...] when I learnt to stay a bit more relaxed, I would say the sensor was the biggest relief.”

## Discussion

The increasing technologization of diabetes treatment over the past 25 years offers opportunities to reduce both the disease burden for those affected and the rising economic costs of treatment. AID systems represent a recent development in diabetes technologies, enabling automated insulin delivery by algorithmically linking CSII and CGM systems. The aim of this study was not only to examine glycemic control outcomes associated with AID use, but also to focus on psychosocial factors that have been largely neglected in previous research. Therefore, the main emphasis was placed on diabetes distress and its dimensions. The present study is one of the few to examine differences between AID users and non-users across the seven dimensions of diabetes distress. Furthermore, it is among the first to apply a mixed methods approach to explore and contextualize these differences, combining quantitative data with follow-up interviews conducted within the same sample. This approach aimed to provide a deeper understanding and interpretation of observed differences in distress dimensions. Importantly, given the high prevalence of CGM use (~94%) in the non-AID group, the comparison in the present study should be interpreted as AID with automated insulin delivery versus CGM-based diabetes management without automation, rather than technology versus no technology use.

In the present study, lower HbA1c levels were observed in the AID group compared to the non-AID group. This finding is consistent with previous research. The between-group difference of approximately 0.5 percentage points exceeds the average difference of 0.2 percentage points reported in the meta-analysis by Godoi et al. [12] and falls within the range of the 0.1 to 0.9 described in a review by Considine and Sherr [13]. A difference of this magnitude is generally considered clinically meaningful, as each 1% increase in HbA1c is associated with a substantially higher risk of micro- and macrovascular complications [29]. This is particularly relevant given the commonly recommended long-term HbA1c target of 7%, as small differences around this threshold may determine whether patients meet or exceed recommended glycemic targets [30]. In the present sample, mean HbA1c in the AID group was below this threshold, whereas it was above 7% in the non-AID group. Therefore, the observed difference of 0.5% may be considered clinically relevant in our population. However, these differences should be interpreted as associations rather than causal effects given the cross-sectional design. It is also possible that self-selection into AID use contributed to these differences, for example through differences in baseline glycemic control, self-management capacity or healthcare access.

Moreover, lower overall diabetes distress was observed in AID users compared to non-users. This pattern is consistent with previous meta-analyses, reviews, and cohort studies [12,13]. The effect size found in the present study (Cohen’s d ≈ 0.6) is larger than effects reported in previous studies [12]. One possible explanation is that clinical studies often recruit subjects with already lower baseline distress levels. Additionally, the outpatient context provides subjects with strong support, which might not be available in other settings, potentially leading to smaller effects than those observed in real-world contexts [13].

As only one comparative study [24] has examined diabetes distress subdimensions, our findings are also discussed in relation to studies on related constructs, such as Hypoglycemia Anxiety [31], as well as findings from cohort studies [23] and qualitative studies on diabetes distress [22]. Consistent with the findings of Kudva et al. [24], lower values in the Powerlessness dimension were observed in the AID group compared to the non-AID group. In contrast to this previous study reporting effects limited to this dimension, our study observed significant differences between AID users and non-users across all sub-dimensions, with the effect size being one of the largest for the Powerlessness dimension. The qualitative part of our study suggests that AID systems may reduce exposure to extreme glycemic fluctuations, which participants linked to reduced feelings of helplessness. In the study by Suttiratana et al. [22], one participant also described diabetes management as a demoralizing daily task, but noted that the use of the system made it less demoralizing for the first time since childhood.

The largest group difference was observed for the Management Distress dimension, with AID users showing a medium-sized advantage. Qualitative data from previous research [22] and the present study suggest that automated insulin delivery may be associated with a reduced cognitive and behavioral burden of diabetes management. For example, AID users described a greater sense of freedom and flexibility in terms of physical activity, which the system enables. This sense of freedom and flexibility, often unfulfilled in daily life, was expressed as a major desire by participants in the non-AID group. A further negative correlation was observed between participants’ age and their distress levels in this diabetes dimension. In the qualitative part of the study, acceptance of one’s illness and age were often jointly discussed. A previous longitudinal study identified acceptance as a significant predictor of reduced diabetes distress over time [32]. Therefore, the observed association may reflect age-related changes in acceptance, although alternative explanations such as disease duration and related adaptation processes cannot be excluded. Importantly, adjusting for age in the ANCOVA models did not materially change group differences.

Moreover, lower Hypoglycemia Distress was observed in the AID group, which is consistent with four of the five studies included in the meta-analysis by Godoi et al. [12]. However, the effect size was smaller than expected. One possible explanation is the widespread use of CGM systems in the non-AID group, which participants described as helpful in managing hypoglycemia. Previous studies have shown that CGM systems are associated with reductions in hypoglycemia anxiety and a lower frequency of hypoglycemic events compared to glucose measurement with test strips [16]. Thus, differences between groups may be attenuated by high baseline levels of technological support in both groups. Nevertheless, AID systems may differ from CGM alone in their ability to actively prevent hypoglycemia events, which may explain the small to moderate group differences and which is illustrated in the following quote: “Well, there were really three worlds in which I lived. I would say that the time before using a CGM system was often characterized by the fact that my wife really had to look after me at night with orange juice and cola to get me out of a hypo, so to speak, or in some cases even ambulance support was necessary. That was super stressful. That’s super stressful. It’s a really stressful situation for a partner or family. The use of a CGM system had reduced this very, very significantly; even when there was the rare case of hypos, there was an alarm. It’s totally annoying, but of course it requires less action than if someone develops massive hypoglycemia and since now, I’ve had the AID system since 2016, there are really only alarm situations in situations where technical failures occur, such as battery failure or sensor errors. Otherwise, the nights go by so that you can really sleep through the night and wake up relaxed the next morning. It really makes such a massive difference; you can’t even imagine it. It’s really incredibly valuable and enriching and I’m very, very grateful.”

As in the other dimensions, significant differences between AID users and non-users were found in the Negative Social Perception sub-dimension, although the effect size was small. This contrasts with the cohort study by Polonsky et al. [23], in which no significant differences were found between baseline and follow-up. Qualitative findings by Suttiratana et al. [22], however, suggest that discretion in everyday life may represent an advantage of AID systems. Participants reported feeling more comfortable managing their diabetes via mobile phone rather than using more visible traditional devices, such as glucose meters or insulin pumps. In the qualitative part of the present study, participants similarly described greater discretion when using diabetes technologies, such as CSII and CGM systems. However, AID systems themselves were not specifically identified as providing additional advantages in this regard. Furthermore, quantitative analyses indicated an effect of age on this dimension. Consistent with this, participants in the qualitative interviews reported experiencing less social stress with increasing age. For example, diabetes devices were worn more openly and less hidden as participants grew older. This finding aligns with the qualitative study by Haynes et al. [33], in which children reported wearing their insulin pumps with pride rather than hiding them as they got older.

Eating distress was also lower in the AID group than in the non-AID group, although the effect size was small. This is partly consistent with the cohort study of Polonsky et al. [23], in which eating distress showed one of the greatest reductions after three months of follow-up. The interview data suggest that perceived reliance on the automated glucose regulation may reduce food-related cognitive burden and concerns about miscalculations. The small effect, observed in the present study should, however, be interpreted with some caution, as the Eating Distress subscale showed slightly lower internal consistency compared to the other subscales, although it remained within an acceptable range for short scales.

Moreover, lower Physician Distress was observed in the AID group. This effect was small to moderate and is consistent with the small improvement observed on this dimension after a three-month follow-up in the cohort study by Polonsky et al. [23]. In the interviews conducted, two types of interactions between AID users and their respective practitioners were identified. The first type of interaction was not considered valuable, but also not stressful, as the interviewees stated that they were able to manage their disease effectively with the support of the system. In the second type of interaction, some participants reported trust and gratitude towards healthcare providers who had encouraged them to adopt the new technologies. This may indicate that the observed association is influenced by relational factors such as perceived support or satisfaction with care, rather than technology use per se. These characteristics have been identified in previous studies as predictors of a good practitioner-patient relationship [34].

In the present study, values on the distress dimension Family and Friends Distress were lower in the AID group than in the non-AID group, whereas no differences were reported by Polonsky et al. [23]. In the interviews, AID users described their social environment as less burdened, referring to fewer extreme hypo- and hyperglycemic events and reduced involvement of others in diabetes management. They further noted that acute glycemic fluctuations were less visible to those around them. This may reflect a reduced salience of the illness in everyday interactions, which in turn may be associated with lower distress, particularly when individuals no longer perceive themselves as a source of stress for others. Tsiouli et al. [35] reported a bidirectional association between family conflicts and suboptimal glycemic control in pediatric type 1 diabetes. In contrast, participants in the AID group did not link this dimension of distress to glycemic control in the qualitative data. This suggests that the current conceptualization of this sub-dimension may not fully capture relevant sources of distress in AID users, warranting further investigation. The concept of shared illness appraisal (i.e., understanding the illness as a shared rather than individual responsibility) may provide a useful framework in this context, as it has been associated with improved well-being for both partners [36]. The expressed desire for greater attention and recognition from family members, which emerged as a pattern in the interviews, aligns with this perspective. Although AID systems may reduce the visibility of diabetes, they do not eliminate its presence.

The obstacles and challenges related to diabetes technologies identified in the qualitative section are consistent with previously reported limitations of CSII and CGM systems. Common CSII-related issues include occlusions and skin irritation [10,37], whereas CGM systems are frequently associated with warm-up phases, signal loss, and occasional and inaccurate or missing glucose values [38]. In the present study, however, such technology-related challenges were not consistently attributable to AID use status or specific device components, but rather reflected general experiences with diabetes technologies across participants. These issues highlight the need for ongoing improvements in diabetes technologies. Addressing such limitations remains an important consideration for manufacturers to support more reliable and less burdensome use. In addition, improving accessibility and reducing barriers to use may be relevant for broader uptake. Improvements in these areas may contribute to enhancing the potential benefits of these technologies for individuals and society.

### Strengths and limitations

The strengths of this study include the relatively large sample size of 169 participants. Moreover, the group sizes of AID users and non-users, as well as the distribution of socio-demographic variables between the groups, were largely equivalent. This enhances the comparability of the groups and strengthens the validity of the results. By focusing on diabetes distress and its specific sub-dimensions, this study also addressed psychosocial factors that have received limited attention in previous research.

The mixed-method design represents a further strength, as it enabled the integration of quantitative and qualitative data. This approach facilitates a more comprehensive interpretation of the findings and allowed for the examination of both statistical associations and participants’ experiences within the same sample. The qualitative component was particularly informative in identifying themes not captured in the quantitative analysis, such as the expressed desire for greater support and attention from friends and family. Notably, this aspect is not reflected in the Family and Friends Distress dimension, which focusses on distress arising from excessive or intrusive attention and control.

A key limitation of this study is the cross-sectional design, which precludes causal inference of the observed associations between AID use, diabetes distress, and glycemic control. In addition, self-selection into AID use may have introduced bias, as individuals were not randomly assigned to treatment conditions. AID users and non-users may differ in characteristics such as health engagement, self-management capacity, access to care, or attitudes toward technology. As these factors are associated with both the likelihood of adopting AID systems and levels of diabetes distress, it remains difficult to disentangle whether observed group differences are attributable to AID use itself or to underlying differences between individuals.

These considerations also have implications for the generalizability of the findings. The sample may not fully represent the broader population of individuals with type 1 diabetes, particularly those who are less engaged in diabetes management or less inclined to use diabetes technologies. This is further reflected in the high prevalence of CGM use in the non-AID group (94%). In Germany, CGM adoption among adults with type 1 diabetes increased from 35% to 75% in 2022 [28], with likely further increases since then, while uptake among children and adolescents has been reported at approximately 95% [39]. Although the level of CGM use observed in the present sample is therefore broadly in line with current trends, it may still limit representativeness with respect to individuals with lower engagement in diabetes technologies. The predominance of female participants in both groups may further restrict generalizability.

HbA1c values were collected via self-reports rather than laboratory measurement, which may introduce measurement error. However, previous studies suggest good agreement between self-reported and laboratory-measured HbA1c. Trivedi et al. [40] reported that 78% of participants with type 2 diabetes accurately reported their HbA1c values, with good intraclass correlation (ICC = 0.84). Similarly, a study conducted in Saudi-Arabia [41] found a mean difference of 0.27% between self-reported and laboratory HbA1c values. In addition, individuals with type 1 diabetes in Germany typically receive regular HbA1c assessments in clinical care, which may support familiarity with their values. While self-reported HbA1c represents a limitation, any reporting bias is likely to be comparable between groups.

Moreover, the qualitative component of this study is subject to several limitations. The relatively small sample size (n = 12) may limit the transferability of the findings and may not fully capture the diversity of experiences among individuals with type 1 diabetes. As participants were recruited from the quantitative sample, the perspectives represented may reflect those of more engaged or technology-affine individuals. Furthermore, the interview guide was developed based on the quantitative findings within an explanatory sequential design, with the aim of better understanding observed and unexpected results. While this approach enabled a focused and in-depth exploration of underlying mechanisms, it may have provided less opportunity for the emergence of entirely new themes. The iterative refinement of the interview guide sought to incorporate emerging topics, although some variability between interviews cannot be excluded. In addition, interviews were conducted using different formats (in person, telephone, and video), which may have influenced the depth and nature of responses. Finally, the qualitative analysis involved interpretative decisions, particularly in the selection and summarization of interview excerpts, their assignment to specific diabetes distress dimensions, and their use in explaining quantitative findings. While the joint display approach enabled a structured integration of both data sources, alternative interpretations of participants’ accounts cannot be fully excluded.

### Outlook and conclusion

The benefits of automated insulin delivery systems in terms of glycemic control have been widely demonstrated in recent years and were also reflected in the present study. However, less is known about their psychosocial effects. By examining differences in diabetes distress and its dimensions, this study suggests that the impact of AID systems extends beyond physical outcomes. Lower level of distress were observed across all dimensions in the AID group compared to the non-AID group.

Given that psychosocial effects of these systems remain insufficiently understood and are closely linked to individuals’ experiences and perceptions of both disease and technologies use, a mixed-methods design was applied. Integrating quantitative results with qualitative interview data enabled a more differentiated interpretation of observed differences, including both anticipated and emergent themes.

Future research should further investigate psychosocial outcomes with current and emerging diabetes technologies, as well as factors influencing their uptake and sustained use. In particular, the role of individual differences in technology exposure (e.g., duration of insulin pump, CGM, or AID use), which was not assessed in the present study, may be relevant for a better understanding of psychosocial outcomes. Moreover, personality traits may warrant further attention, as these have previously been associated with glycemic control and self-management and may act as relevant moderating or mediating factors [42].

AID systems may contribute to more individualized diabetes care due to their adaptability to users’ needs. A better understanding of factors influencing technology adaption, as well as patient-technology interactions, may support more patient-centered implementation and use of these systems.
